# Sociodemographic Disparities in Sodium-Glucose Co-Transporter-2 Inhibitors and Glucagon-Like Peptide-1 Receptor Agonists Prescription Patterns Among Patients With Poorly Controlled Diabetes

**DOI:** 10.7759/cureus.56845

**Published:** 2024-03-24

**Authors:** Daniel Antwi-Amoabeng, Bryce D Beutler, Jasmine Ghuman, Mark B Ulanja, Joban Ghuman, Nageshwara Gullapalli

**Affiliations:** 1 Internal Medicine, Christus Ochsner St. Patrick Hospital, Lake Charles, USA; 2 Radiology, University of Southern California Keck School of Medicine, Los Angeles, USA; 3 Internal Medicine, University of Nevada Reno School of Medicine, Reno, USA; 4 Cardiology, University of Utah Health, Salt Lake City, USA

**Keywords:** mortality, sodium glucose co-transporter-2 inhibitors, sglt inhibitors, racial disparity, glucagon-like peptide-1 receptor agonists, glp-1 agonists

## Abstract

Introduction

Sodium-glucose co-transporter-2 inhibitors (SGLT2Is) and glucagon-like peptide-1 receptor agonists (GLP-1RAs) are novel antihyperglycemic agents that reduce cardiovascular mortality through insulin-independent mechanisms. In this cross-sectional study, we investigated prescription patterns of these drugs and identified inequities in antihyperglycemic utilization.

Methods

Unique encounters for diabetes care between January 1, 2020, and December 31, 2020, were identified through a systematic query of our healthcare system’s database. All patients ≥18 years old with a hemoglobin A1C level of ≥8% were included in the sample. Demographic data, SGLT2I or GLP-1RA prescription status, diabetes-related complications, and mortality were abstracted.

Results

A total of 2,746 patients were included in the sample. Among these individuals, 670 (24.4%) were prescribed either an SGLT2I or a GLP-1RA (users) and 2,076 (75.6%) were not prescribed either agent (non-users). There were significantly more males than females in the cohort, but there was no significant difference in the sex distribution between users and non-users. Compared to non-users, users were younger (mean age of 65.1 ± 9.4 years versus 66.4 ± 9.9 years, p-value = 0.005), more likely to be non-Hispanic (86.3% versus 13.7%), more likely to live in a middle-income zip code, and have private insurance. The mortality rate was lower among users when compared to non-users, but the difference did not reach statistical significance (2.7% versus 5.5%, p-value = 0.62). SGLT2I use was associated with a 60% lower risk of mortality.

Conclusion

Ethnicity, median household income, and insurance type influence the likelihood of being prescribed an SGLT2I or a GLP-1RA. Individuals prescribed either agent appear to have better mortality outcomes than those prescribed other medications. Further investigation may reveal underlying causes and potential solutions for disparities in prescription patterns.

## Introduction

Sodium-glucose co-transporter-2 inhibitors (SGLT2Is) and glucagon-like peptide-1 receptor agonists (GLP-1RAs) are antihyperglycemic agents that were approved by the U.S. Food and Drug Administration (FDA) in 2013 and 2012, respectively, for the treatment of non-insulin-dependent diabetes mellitus. Data suggest that these therapies reduce hemoglobin A1C (HbA1C), facilitate weight loss, and reduce cardiovascular mortality through insulin-independent mechanisms [1,2]. In addition, emerging evidence indicates that both SGLT2Is and GLP-1RAs may decrease all-cause mortality [3,4]. Both medication classes are generally well-tolerated with relatively few adverse effects [5,6]. However, despite the proven benefits of SGLT2Is and GLP-1RAs, their use is limited by cost.

Racial, ethnic, and socioeconomic disparities in the pharmacologic management of diabetes mellitus have previously been described [7,8], and recent data suggest that racial minorities are less likely to be prescribed SGLT2Is and GLP-1RAs as compared to their White counterparts [9]. In this cross-sectional study, we aimed to investigate the association between sociodemographic characteristics (including race, ethnicity, sex, income, and insurance status) and the likelihood of receiving a prescription for an SGLT2I or GLP-1RA. We further assessed the influence of these covariables on all-cause mortality of the subjects. Identifying the barriers to the use of medications with proven mortality benefits using data readily available in electronic medical records may allow hospital systems to appropriately target medication affordability programs for at-risk individuals.

A portion of this article was presented virtually as a meeting abstract at the 2021 Scientific Session of the American Heart Association.

## Materials and methods

Study subjects

We performed a systematic query of our healthcare system database using International Classification of Diseases 10th edition (ICD-10) codes for diabetes mellitus type 2 for all care encounters between January 1, 2020, and December 31, 2020. Care encounters included emergency room visits and hospitalization at either of the hospitals in our 946-bed tertiary care hospital system in Northern Nevada, USA, or primary care or urgent care visits within the system. All patients who were at least 18 years old, with HbA1C ≥8%, and received at least a single prescription for SGLT2I or GLP-1RA during the study period were included in the study. We chose this HbA1c level, which has been used previously [10], to capture a sample of subjects with poorly controlled diabetes in whom SGLT2I and/or GLP-1RA would be a reasonable hyperglycemia therapy based on the recommendation by the American Diabetes Association to target HbA1c below 8% for a large portion of patients irrespective of the presence of multiple comorbidities, a history of hypoglycemia, or advanced diabetes complications [11].

Comorbid conditions and demographic data, including race, ethnicity, zip code of residential address, mortality rate, and cause of death, were abstracted from reviewing individual charts. Race and ethnicity were defined based on self-identification as reported in the medical record. Residential zip code was used as a surrogate for median annual household income [12,13]. Cause of death was as documented in the death summary or note available in the medical record at the time of data abstraction. For every subject with at least one prescription for either SGLT2I or GLP-1RA (users), we planned to include two subjects without a record of either prescription (non-user). Our sample-size determination calculations showed that 687 non-users and 344 users would be needed to detect a difference of approximately 18.9 deaths per 100,000 population with a power of 80% at a 5% level of significance. The study was conducted as part of a quality improvement project at our institution.

Statistical analysis

Baseline characteristics of the subjects were summarized as mean ± standard deviation or proportions. The frequency distribution of comorbid conditions and mortality outcomes were compared using the chi-square test of independence. The Wilcoxon rank-sum test or Student’s t-test was used to compare age and household income between users and non-users, as applicable. We constructed a multivariable logistic regression model to assess the factors that influence medication prescription status and mortality. Model specification and fit were assessed by the linktest command and Hosmer-Lemeshow goodness-of-fit test, respectively. Model selection was adjudicated based on the Akaike Information Criterion (AIC). An exploratory analysis using race (with all categories included) did not yield significant results, and those models had poor fit and specification, likely due to the small number of observations. Thus, race was excluded from the final model. All analyses were performed at two-tailed 5% level of significance using Stata version 16.1 (Stata Corp., College Station, TX).

## Results

We included 2,746 patients in our study. Prescriptions for GLP-1RAs and/or SGLT2Is medications were present in 24.4% of the patients (users). Males were overrepresented in the sample, but there was no significant difference in sex distribution between users and non-users. The mean age of users was 65.1 ± 9.4 years, which was significantly younger than non-users at 66.4 ± 9.9 years (p = 0.005). Subgroup analysis revealed that age did not influence prescription of individual medications. Among users, significantly more patients identified as White (77.9%) as compared to other racial groups, including Black, Asian, Native American, and Pacific Islander. Non-Hispanic was the largest ethnic group in our sample (86.3% versus 13.7%). Using zip code as a surrogate for income level, we found that users were more likely to reside in areas with a median annual household income in the middle-income category based on 2018 income tiers ($48,500-$145,500). Users were significantly more likely than non-users to have private insurance (p<0.001). Medicaid was predictive of SGLT2I but not GLP-1RA use, whereas private insurance was associated with GP1-RA use only. Table 1 summarizes the baseline subject characteristics based on medication use status.

**Table 1:** Baseline comparison of patient characteristics by prescription status. “Other race” was not defined in our database, and “unknown race” refers to those observations with missing race data. GLP-1RA, glucagon-like peptide-1 receptor agonists; SGLT2I, sodium-glucose co-transporter-2 inhibitors

	SGLT2I and/or GLP-1RA		P-value
	Non-users, 2,076 (75.6%)	Users, 670 (24.4%)	
Mean age ± SD	66.4 ± 9.9	65.1 ± 9.4	0.005
Median household income (US dollars)	52,818	56,326	<0.0001
	n (%)	n (%)	
Age categories			0.03
<65 years	971 (46.8)	346 (51.6)	
≥65 years	1,105 (53.2)	324 (48.4)	
Sex			0.15
Female	929 (44.8)	321 (47.9)	
Male	1,147 (55.2)	349 (52.1)	
Ethnicity			<0.001
Hispanic	431 (20.8)	92 (13.7)	
Non-Hispanic	1,645 (79.2)	578 (86.3)	
Race			0.28
White	1,579 (76.1)	522 (77.9)	
Black	63 (3)	24 (3.6)	
Asian	125 (6)	46 (6.9)	
Native Hawaiian or Other Pacific Islander	23 (1.1)	8 (1.2)	
American Indian or Alaska Native	51 (2.5)	17 (2.5)	
Other race	38 (1.8)	11 (1.6)	
Unknown	197 (9.5)	42 (6.3)	
Income class			<0.001
Low income	858 (41.3)	202 (30.2)	
Middle income	1,217 (58.7)	467 (69.8)	
Insurance type			<0.001
Medicare	1,037 (50)	283 (42.2)	
Medicaid	127 (6.1)	20 (3)	
Private	585 (28.2)	244 (36.4)	
Other	63 (3)	12 (1.8)	
Unknown	264 (12.7)	111 (16.6)	

There were no significant differences in the proportion of specific diabetes complications between users and non-users (Table 2).

**Table 2:** Comparison of diabetes complications and mortality by prescription status. Unknown cause of death refers to those who died outside of the hospital, and other cause of death refers to causes other than those stated. DKA, diabetic ketoacidosis; GLP-1RA, glucagon-like peptide-1 receptor agonists; SGLT2I, sodium-glucose co-transporter-2 inhibitors

	SGLT2I and/or GLP-1RA		P-value
	Non-users, n (%)	Users, n (%)	
	2,076 (75.6)	670 (24.4)	
Diabetes-related complications			
DKA without coma	65 (3.1)	9 (1.3)	0.76
Chronic kidney disease	144 (6.9)	46 (6.9)	1
Other kidney complication	44 (2.1)	26 (3.9)	0.66
Retinopathy	13 (0.6)	3 (0.5)	0.98
Neuropathy	75 (3.6)	20 (3)	0.9
Peripheral artery disease without gangrene	22 (1.1)	15 (2.2)	0.79
Foot ulcer	39 (1.9)	8 (1.2)	0.89
No complications	1,674 (80.6)	543 (81)	0.84
Mortality			
Dead	114 (5.5)	18 (2.7)	0.61
Alive	1,962 (94.5)	652 (97.3)	0.004
Cause of death			
COVID-19	35 (30.7)	5 (27.8)	0.89
Sepsis	20 (17.5)	1 (5.6)	0.76
Stroke	15 (13.2)	3 (16.7)	0.87
Cardiovascular	10 (8.8)	3 (16.7)	0.7
Cancer	9 (7.9)	3 (16.7)	0.66
Other	14 (12.3)	0	-
Unknown	11 (9.6)	3 (16.7)	0.72

**Figure 1:**
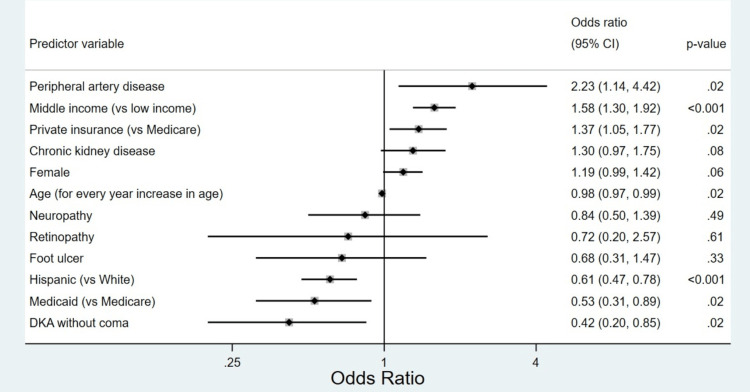
A forest plot showing adjusted odds ratios of the predictors of SGLT2I and/or GLP-1RA prescription in the cohort. DKA, diabetic ketoacidosis; GLP-1RA, glucagon-like peptide-1 receptor agonists; SGLT2I, sodium-glucose co-transporter-2 inhibitors

Among diabetes-related complications, only peripheral artery disease (PAD) and a history of diabetic ketoacidosis (DKA) had significant influence on the odds of receiving a prescription. History of PAD or chronic kidney disease was associated with significantly increased odds of receiving a prescription for a GLP-1RA but not SGLT2I (data not shown). Notably, this observation may be explained by the fact that an estimated glomerular filtration rate (eGFR) of less than 30 mL/min/1.73 m2 represents a contraindication for SGLT2I use. Older age, Hispanic ethnicity, Medicaid insurance, and a history of DKA were associated with significantly lower odds of being prescribed an SGLT2I and/or GLP-1RA (Figure 1).

**Figure 2:**
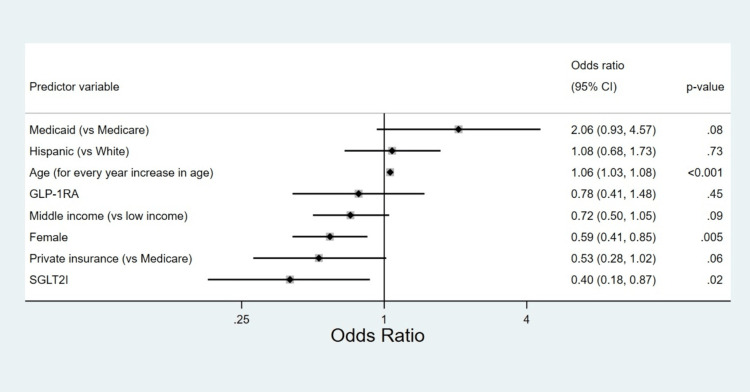
A forest plot demonstrating the predictors of all-cause mortality based on the multivariable logistic regression model. GLP-1RA, glucagon-like peptide-1 receptor agonists; SGLT2I, sodium-glucose co-transporter-2 inhibitors

Between January 2020, and June 2021, a total of 132 patients in our study population died. The all-cause mortality rate was 2.7% among users of SGLT2I/GLP-1RA as compared to 5.5% in non-users. The difference in the proportion of patients who died was not statistically significant between the two groups (p = 0.6). The adjusted odds of mortality were significantly lower among SGLT2I users as compared to non-users and in females compared to males (Figure 2). A multivariable logistic regression model showed that age was associated with a significant increase in the odds of death (adjusted odds ratio of 1.06; 95% CI: 1.03-1.08; p-value <0.001). There was no significant difference in the distribution of the cause of death between users and non-users.

## Discussion

SGLT2Is and GLP-1RAs are safe, well-tolerated, and highly effective hypoglycemic agents that have been shown to reduce all-cause mortality among patients with non-insulin dependent diabetes mellitus. However, despite the proven benefits of these novel medications, prescription rates remain relatively low [14]. Our analysis reveals significant inequity in SGLT2I and GLP-1RA prescriptions affecting racial minorities and low-income individuals.

We found that individuals who identified as Hispanic were significantly less likely to receive a prescription for an SGLT2I and/or GLP-1RA as compared to their non-Hispanic counterparts. Healthcare inequity affecting the Hispanic community is not without precedent. Previous studies have demonstrated that Hispanic individuals with cardiovascular risk factors, including hypertension and hypercholesterolemia, are often undertreated [15-17]. Furthermore, as compared to non-Hispanic Whites, Hispanic patients are more likely to demonstrate medication nonadherence [18] and less likely to fill prescriptions [19]. It has been postulated that inadequate access to care [20], patient-physician language barriers [21], prohibitive costs [22,23], and distrust of the healthcare system contribute to treatment inequities among the Hispanic population. However, irrespective of the underlying causes, inadequate treatment in the setting of nonadherence can have a devastating effect on health outcomes. Given the increased risk of nonadherence associated with complex treatment regimens, SGLT2Is and GLP-1RAs with one daily and once weekly forms should be considered mainstay therapies among the Hispanic community. Nevertheless, our data demonstrate that eligible Hispanic individuals are significantly less likely than non-Hispanic Whites to be prescribed an SGLT2I or GLP-1RA.

Disparities in prescription patterns are not limited to ethnicity; our analysis suggests that individuals residing in low-income communities are less likely to be prescribed an SGLT2I or GLP-1RA than those living in middle- or higher-income areas. These findings are consistent with other investigations of prescription patterns for high-cost medications. In a study by Inselman et al., for example, authors found that low-income individuals with asthma were less likely to be prescribed new biologics as compared to their high-income counterparts [24]. Low-income patients are also less likely to be prescribed opioid medication for pain control [25] and are at an increased risk of early antidepressant discontinuation [26] as compared to those in higher economic strata; although these drugs are typically inexpensive, the findings highlight existing prescription disparities between low- and high-income patients.

Prescription pattern disparities based on income are likely related to multiple underlying factors. Clinicians may be reluctant to recommend high-priced medications to low-income patients with diabetes, as these individuals already face excessive healthcare costs even when using generic medications [27]. Bias may also play a role: low-income patients are often perceived as non-adherent and thus ostensibly less likely to benefit from high-cost medications [28]. Moreover, it has been established that expensive prescriptions are less likely to be filled than low-cost alternatives [29], which may prompt clinicians to recommend generic medications. Improving outcomes among low-income patients with diabetes may therefore require a multi-pronged approach that includes shared decision-making, fee-assistance programs, and implementation of psychosocial strategies known to improve medication adherence [30].

There were several limitations of the study inherent to its design. Data were collected from a single institution and thus the findings cannot be generalized. Mortality events that occur outside of our healthcare system are not routinely captured unless a primary care physician enters documentation in the chart. Therefore, mortality rates presented here may be underestimated. Furthermore, specific patient characteristics, such as co-administered medications, discontinuation of SGLT2I/GLP-1RA therapy, or switching from one to the other, and specific causes of mortality, were not abstracted; thus, allocation bias could not be definitively excluded. Although all subjects in this study had an HbA1c level of ≥8%, it is reasonable to expect that treating HbA1c as a continuous variable may have reviewed nuanced prescription patterns as clinicians are more likely to adopt alternative therapies in order to attain HbA1c targets. We combined the prescription rates for both classes of medication as a minority of patients were on both and could not be excluded from the analysis. However, owing to the recent rise in the popularity of GLP-1RAs for their weight loss benefits and the media coverage of rising prices, an analysis assessing each class of medication separately may be more informative. These limitations notwithstanding, our study demonstrates that socioeconomic factors play a significant role in the prescription pattern of SGLT2I and GLP-1RA drugs among patients with poorly controlled diabetes.

## Conclusions

In this cross-sectional study, we show that low-income individuals are significantly less likely to receive a prescription for SGLT2Is and/or GLP-1RAs as compared to their higher income counterparts. Like other high-cost drugs, we also identified significant ethnic disparities in SGLT2I and GLP-1RA prescription patterns; users were more likely to be White. Our analysis suggests that the high cost of drugs may discourage clinicians from prescribing these antidiabetic agents despite their proven benefit on cardiovascular and all-cause mortality among patients with non-insulin dependent diabetes mellitus who meet indication but are economically disadvantaged. Further investigation to better understand the underlying causes of economic and ethnic disparities in SGLT2I and GLP-1RA prescription rates may help improve outcomes for all eligible patients with non-insulin dependent diabetes mellitus.

## References

[1] Inzucchi SE, Zinman B, Wanner C (2015). SGLT-2 inhibitors and cardiovascular risk: proposed pathways and review of ongoing outcome trials. Diab Vasc Dis Res.

[2] Bucheit JD, Pamulapati LG, Carter N, Malloy K, Dixon DL, Sisson EM (2020). Oral semaglutide: a review of the first oral glucagon-like peptide 1 receptor agonist. Diabetes Technol Ther.

[3] Silverii GA, Monami M, Mannucci E (2021). Sodium-glucose co-transporter-2 inhibitors and all-cause mortality: a meta-analysis of randomized controlled trials. Diabetes Obes Metab.

[4] Rasmussen MF (2020). The development of oral semaglutide, an oral GLP-1 analog, for the treatment of type 2 diabetes. Diabetol Int.

[5] Saisho Y (2020). SGLT2 inhibitors: the star in the treatment of type 2 diabetes?. Diseases.

[6] Granhall C, Donsmark M, Blicher TM, Golor G, Søndergaard FL, Thomsen M, Bækdal TA (2019). Safety and pharmacokinetics of single and multiple ascending doses of the novel oral human GLP-1 analogue, oral semaglutide, in healthy subjects and subjects with type 2 diabetes. Clin Pharmacokinet.

[7] Allsworth JE, Toppa R, Palin NC, Lapane KL (2005). Racial and ethnic disparities in the pharmacologic management of diabetes mellitus among long-term care facility residents. Ethn Dis.

[8] Eberly LA, Yang L, Eneanya ND (2021). Association of race/ethnicity, gender, and socioeconomic status with sodium-glucose cotransporter 2 inhibitor use among patients with diabetes in the US. JAMA Netw Open.

[9] Whyte MB, Hinton W, McGovern A (2019). Disparities in glycaemic control, monitoring, and treatment of type 2 diabetes in England: a retrospective cohort analysis. PLoS Med.

[10] Crowley MJ, Holleman R, Klamerus ML, Bosworth HB, Edelman D, Heisler M (2014). Factors associated with persistent poorly controlled diabetes mellitus: clues to improving management in patients with resistant poor control. Chronic Illn.

[11] (2013). Standards of medical care in diabetes--2013. Diabetes Care.

[12] (2024). https://www.unitedstateszipcodes.org/..

[13] Bennett J, Fry R, Kochhar R (2024). Are you in the American middle class? Find out with our income calculator. Pew Research Center. https://www.pewresearch.org/short-reads/2020/07/23/are-you-in-the-american-middle-class/.

[14] Gay H, Yu J, Petito L (2020). Abstract 14678: Prevalence Of Sglt2 inhibitor and Glp-1 receptor agonist prescriptions in patients with comorbid diabetes and cardiovascular disease in an integrated academic health system. Circulation.

[15] Gu A, Yue Y, Desai RP, Argulian E (2017). Racial and ethnic differences in antihypertensive medication use and blood pressure control among US adults with hypertension: the National Health and Nutrition Examination Survey, 2003 to 2012. Circ Cardiovasc Qual Outcomes.

[16] Qato DM, Lindau ST, Conti RM, Schumm LP, Alexander GC (2010). Racial and ethnic disparities in cardiovascular medication use among older adults in the United States. Pharmacoepidemiol Drug Saf.

[17] Mann D, Reynolds K, Smith D, Muntner P (2008). Trends in statin use and low-density lipoprotein cholesterol levels among US adults: impact of the 2001 National Cholesterol Education Program guidelines. Ann Pharmacother.

[18] Fernández A, Quan J, Moffet H, Parker MM, Schillinger D, Karter AJ (2017). Adherence to newly prescribed diabetes medications among insured Latino and White patients with diabetes. JAMA Intern Med.

[19] Reed M, Hargraves JL (2003). Prescription drug access disparities among working-age Americans. Issue Brief Cent Stud Health Syst Change.

[20] Burner E, Terp S, Lam CN, Neill E, Menchine M, Arora S (2019). Access to care, nativity and disease management among Latinos with diabetes in a safety-net healthcare setting. AIMS Public Health.

[21] Fernandez A, Schillinger D, Warton EM (2011). Language barriers, physician-patient language concordance, and glycemic control among insured Latinos with diabetes: the Diabetes Study of Northern California (DISTANCE). J Gen Intern Med.

[22] Lyles CR, Seligman HK, Parker MM (2016). Financial strain and medication adherence among diabetes patients in an integrated health care delivery system: the Diabetes Study of Northern California (DISTANCE). Health Serv Res.

[23] Thomas A, Ashcraft AS, Owen DC, Conway-Phillips R (2017). Making it all work: qualitative descriptions of Hispanic adults managing type 2 diabetes with limited resources. Glob Qual Nurs Res.

[24] Inselman JW, Jeffery MM, Maddux JT, Shah ND, Rank MA (2020). Trends and disparities in asthma biologic use in the United States. J Allergy Clin Immunol Pract.

[25] Joynt M, Train MK, Robbins BW, Halterman JS, Caiola E, Fortuna RJ (2013). The impact of neighborhood socioeconomic status and race on the prescribing of opioids in emergency departments throughout the United States. J Gen Intern Med.

[26] Bocquier A, Cortaredona S, Verdoux H, Casanova L, Sciortino V, Nauleau S, Verger P (2014). Social inequalities in early antidepressant discontinuation. Psychiatr Serv.

[27] Sarpong EM, Bernard DM, Miller GE (2012). Changes in pharmaceutical treatment of diabetes and family financial burdens. Med Care Res Rev.

[28] Willems SJ, Swinnen W, De Maeseneer JM (2005). The GP's perception of poverty: a qualitative study. Fam Pract.

[29] Tamblyn R, Eguale T, Huang A, Winslade N, Doran P (2014). The incidence and determinants of primary nonadherence with prescribed medication in primary care: a cohort study. Ann Intern Med.

[30] Wilhelmsen NC, Eriksson T (2019). Medication adherence interventions and outcomes: an overview of systematic reviews. Eur J Hosp Pharm.

